# The role of probiotics in adolescents’ obesity

**DOI:** 10.3389/fcimb.2025.1546627

**Published:** 2025-07-02

**Authors:** Xiao-ping Chen, Li You, Yong Jia

**Affiliations:** College of Physical Education and Health, Chongqing College of International Business and Economics, Chongqing, China

**Keywords:** probiotics, obesity, adolescence, gut microbiota, lipid metabolism

## Abstract

The prevalence of adolescent obesity continues to rise globally, posing significant public health challenges by affecting both physical and psychological wellness and increasing the risk of metabolic diseases in adulthood. Probiotics may influence obesity through various mechanisms, including restoring gut microbiota balance, reducing chronic inflammation, modulating lipid metabolism, aiding in weight control, and improving metabolic health. This review aims to explore the mechanisms by which probiotics act as key modulators of obesity and summarize current findings from clinical trials involving probiotics in adolescent obesity. The large-scale, multicenter, long-term follow-up randomized controlled trials are necessary to determine the optimal probiotics strains, dosages, and treatment durations, as well as to assess their long-term efficacy and safety in the future. Through such rigorous studies, probiotics have the potential to become a safe, effective, and accessible adjunct in the comprehensive management of adolescent obesity, offering a more holistic approach to health management for this population.

## 1 Introduction

Adolescent obesity has emerged as a significant global public health issue (Morgado et al., 2023). This rise is primarily driven by lifestyle changes and the widespread availability of high-calorie diets, leading to a steady increase in obesity prevalence among adolescents (NCD-RisC, 2017). Importantly, adolescent obesity often persists into adulthood, thereby elevating the lifelong risk of obesity-related diseases (Buscot et al., 2018; Horesh et al., 2021). Obesity is a condition resulting from an imbalance between energy intake and metabolism (Calcaterra et al., 2023). Experimental evidence confirms its impact on health, showing that obesity leads to increased triglyceride and cholesterol levels, which contribute to cardiovascular diseases (Khan et al., 2018; Dikaiou et al., 2021). In recent years, evidence supporting the role of gut microbiota in metabolic diseases has emerged, and the use of prebiotics, probiotics, synbiotics, and postbiotics to modulate gut microbiota has gained popularity (Luzzi et al., 2024). Furthermore, severe obesity can also lead to the development of type 2 diabetes (Lin et al., 2016). Additionally, it can cause fat accumulation in the liver, leading to fatty liver disease, which may progress to fibrosis in severe cases (Caussy et al., 2018). Consequently, the effective prevention and treatment of adolescent obesity have become critical challenges in the fields of medicine and public health.

Probiotics, which are live microorganisms beneficial to host health, have gained considerable attention in recent years (Senok et al., 2005; Mijangos-Trejo et al., 2023).

There are over 500 different microorganisms residing in the normal human gut, numbering in the tens of trillions. In the vast majority of cases, they constrain and coexist with each other. However, toxins produced by some bacteria and viruses, such as cholera toxin, heat labile toxin (LT) and heat stable toxin (ST) of Escherichia coli, can cause acute gastroenteritis and diarrhea. Currently, 7%-10% of the world’s population is troubled by irritable bowel syndrome, and excellent probiotic strains without any toxic side effects are a good choice for treating this disease.

Probiotics are mainly divided into three categories, namely Bifidobacterium, Lactobacillus, and facultative anaerobic cocci. Bifidobacterium genus: There are currently 32 species in this genus, of which only 5 are allowed to be used as probiotics in the human gut, namely Bifidobacterium longum, Bifidobacterium infantis, Bifidobacterium pubertae, Bifidobacterium bifidum, and Bifidobacterium brevis. Lactobacillus genus: There are currently 56 species in this genus, and the main ones with good clinical records include Lactobacillus rhamnosus GG, Lactobacillus casei, and Lactobacillus Johnson L-1. Among them, there are mainly 10 types used for producing microecological agents, including Lactobacillus acidophilus, Lactobacillus brevis, Lactobacillus casei, and Lactobacillus bulgaricus. Facultative anaerobic cocci: including Enterococcus faecalis, Lactococcus lactis, Streptococcus mutans thermophilic subspecies, and Streptococcus mutans, etc (Wang et al., 2013).

Meanwhile, probiotics are widely used in the market, including dairy products, beverages, and food additives. At present, the most common probiotic products on the market are dairy products, with common bacterial strains including Lactobacillus acidophilus, Lactobacillus bulgaricus, Bifidobacterium, etc., which are also added to milk powder or yogurt. Adding probiotics to dairy products has better nutritional and health benefits, regulating the gut microbiota to varying degrees, improving immunity and digestive function, such as absorption of minerals and vitamins, and reduction of fat. At the same time, more and more beverages sold on the market are adding probiotics, such as juiced fermented apples, dragon fruit, carrots, etc. They can enhance the vitality of lactobacillus cells, and vegetables have a better taste after fermentation. The lactic acid obtained can enhance its nutritional value, reduce the content of aflatoxin in lactic acid, and ensure the safety of lactic acid beverage products. At the same time, with the development of food science and technology, probiotics are often added to food additives, which can reduce the content of spices and essence in food and increase the consistency of food. For example, adding probiotic food additives to dairy products can produce products with better flavor and higher consumer preference for probiotics (Ni and Xu, 2024).

Their primary function is to regulate gut microbiota and maintain intestinal ecological balance, thereby promoting digestion, enhancing immunity, and preventing various diseases (Byndloss et al., 2018; Ezzaidi et al., 2019). Recent studies further indicate that gut microbiota plays a crucial role in human energy metabolism and fat storage, with dysbiosis potentially being a key factor in obesity (Miyamoto et al., 2019; Xu et al., 2019). Supporting this, *in vivo* studies have demonstrated that probiotics improve gut microbiota dysbiosis, thereby alleviating obesity in mice (Kong et al., 2019; Sun et al., 2020). Additionally, the probiotics may enhance gut barrier function and reduce inflammatory responses, thereby mitigating obesity-related chronic inflammation (Liu et al., 2020). Studies in mouse models have demonstrated that specific *Bifidobacterium* strain influences obesity by reducing total serum cholesterol, decreasing pro-inflammatory cytokines, and enhancing glucose tolerance (Moya-Pérez et al., 2015). Moreover, probiotics may influence lipid metabolism pathways, decreasing adipogenesis and aiding in weight control (He and You, 2020; Cao et al., 2024).

While animal and some population studies indicate potential benefits of probiotics, their effectiveness in treating adolescent obesity remains uncertain, with most research primarily focused on adults. In randomized controlled trials (RCTs), some studies have demonstrated probiotics can lead to improvements in various health metrics, including anthropometric measurements (such as body weight and BMI), glucose metabolism, and lipid profiles, while in a study involving obese Hispanic adolescents, it was found that probiotic supplementation with VSL#3, delivered through non-caloric, sugar-free flavored drinks (vitamin water zero), was associated with an increase in obesity measures. Furthermore, there were no significant changes in liver fat or gut microbiota composition, indicating potential adverse effects in this specific population (Jones et al., 2018; Yildirim et al., 2022). Therefore, further research and additional clinical trials are required to comprehensively evaluate the role and underlying mechanisms of probiotic interventions in addressing adolescent obesity.

**Figure 1:**
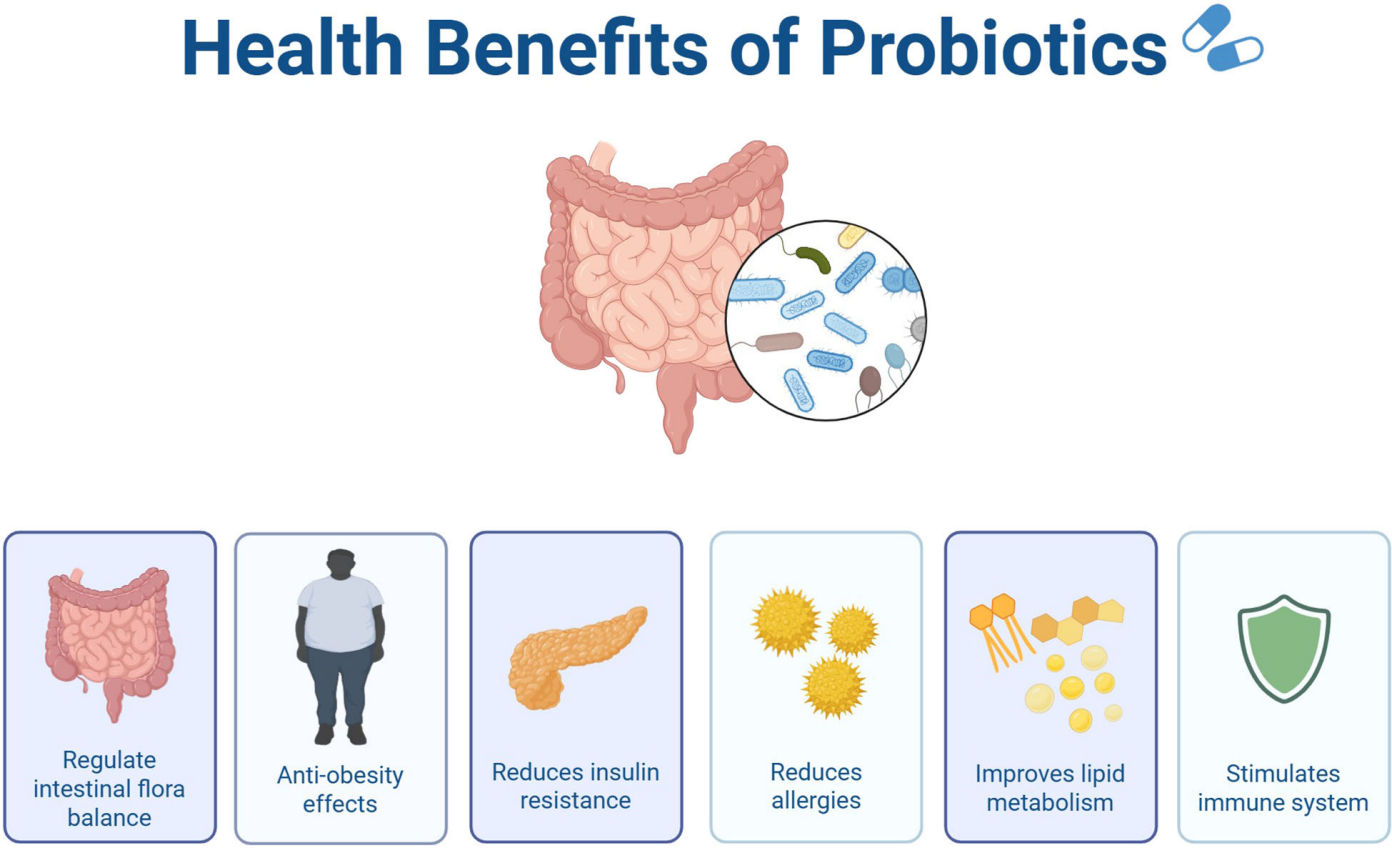
Benefits of probiotics on obesity.

This review aims to examine recent evidence on the role of probiotic interventions in obesity and to summarize findings from studies on probiotics in adolescent obesity. We believe large-scale, long-term clinical studies may provide more robust scientific evidence for incorporating probiotics into comprehensive treatment strategies for adolescent obesity (**Figure 1**).

## 2 The mechanism of probiotics on obesity

### 2.1 Regulation of intestinal flora balance

Dysbiosis of the gut microbiota is strongly associated with obesity, primarily characterized by reduced bacterial richness and diversity (Liu et al., 2017; Gao et al., 2018). While dysbiosis is a significant factor, obesity is a multifactorial condition influenced by various factors, and improving the composition and diversity of the gut microbiota may help in its prevention or treatment.

#### 2.1.1 Human studies

A multi-strain probiotic mixture has been shown to modulate obesity-associated gut microbiota dysbiosis and enhance lipid metabolism in obese children (Chen et al., 2022). A double-blind, randomized, placebo-controlled trial was performed on obese children, who underwent a 12-week probiotic supplementation, combined with dietary and exercise counseling (Chen et al., 2022). The administration of multi-strain probiotics led to an increase in high-density lipoprotein cholesterol (HDL-C) and adiponectin levels, while reductions were observed in body mass index (BMI), serum total cholesterol, low-density lipoprotein-C (LDL-C), leptin, and tumor necrosis factor-alpha (TNF-α) (Chen et al., 2022). The results indicate that multi-strain probiotic supplementation may alleviate gut dysbiosis associated with obesity, offering potential benefits for weight management and health in overweight and obese children (Chen et al., 2022).

A cross-sectional study of Korean children found a link between the gut microbiota and pediatric obesity (Shin and Cho, 2020). Recent research has shown that factors influencing infant overgrowth may be mediated by gut microbiota profiles (Ihekweazu and Versalovic, 2018). A prospective study over a 4-year period identified microbial changes associated with weight gain in children, suggesting that the microbiota–host–diet configuration could predict obesity (Rampelli et al., 2018). Additionally, another study involving Korean children demonstrated that a 2-month weight reduction program led to changes in the composition and function of the gut microbiota in the obese group (Cho, 2021).

#### 2.1.2 Animal studies

Research using animal models provides critical insights into the mechanisms by which probiotics may influence obesity and related metabolic disorders. For instance, *Lactobacillus acidophilus* has been shown to mitigate obesity in mice by restoring gut microbiota balance and enhancing intestinal barrier function (Kang et al., 2022). This probiotic reduces body weight, fat mass, inflammation, and insulin resistance in obese mice while activating brown adipose tissue, thus improving energy expenditure, glucose homeostasis, and lipid metabolism (Kang et al., 2022). Additionally, it restores gut dysbiosis by reducing the *Firmicutes/Bacteroidetes* ratio, maintaining gut barrier integrity, and lowering metabolic endotoxemia (Kang et al., 2022). These findings suggest that *Lactobacillus acidophilus* may alleviate obesity and related conditions such as hyperlipidemia, non-alcoholic fatty liver disease, and insulin resistance by mitigating inflammation, endothelial dysfunction, and gut dysbiosis.

Similarly, *Lactobacillus fermentum* can counteract obesity in mice induced by a high-fat diet by modulating gut microbiota dysbiosis. Its anti-obesity effects are linked to its anti-inflammatory properties and the improvement of endothelial dysfunction and gut microbiota imbalance. This probiotic increases *Akkermansia* levels, decreases *Erysipelotrichaceae* and *Clostridium* populations, and elevates the proportion of *Bacteroides*, thereby alleviating experimental obesity through microbiome modulation (Molina-Tijeras et al., 2021).

Another study demonstrated that probiotics can reduce fat accumulation in obese mouse models by altering the composition of the gut microbiota. Administration of *Lactobacillus plantarum* led to significant reductions in body weight, mesenteric and subcutaneous fat tissues, and liver weight in obese. Furthermore, probiotic intervention decreased serum triglyceride levels and increased HDL-C levels in these mice. Gut microbiota analysis revealed that *Lactobacillus plantarum* treatment reduced the *Firmicutes/Bacteroidetes* ratio, indicating an improvement in gut microbiota composition. This intervention also elevated total short-chain fatty acid (SCFA) levels, significantly reducing fat content in both adipose tissue and liver, alongside improved gene expression related to lipid metabolism, adipogenesis, and SCFA receptors (Joung et al., 2021). Overall, obesity is closely linked to lipid accumulation and gut microbiota dysbiosis. These studies demonstrate that probiotic supplementation may alleviate obesity symptoms by restoring gut microbiota balance.

### 2.2 Regulation of lipid metabolism

Obesity is distinctly characterized by abnormal lipid accumulation in the body and is frequently associated with hyperlipidemia, marked by elevated circulating triglyceride and free fatty acid levels (Jain et al., 2007). An increase in both the number and size of adipocytes is a hallmark of obesity, closely linked to adipose tissue dysfunction. To date, mounting evidence indicates that probiotics serve as anti-obesity agents by enhancing lipid metabolism. *Bifidobacterium lactis* has demonstrated anti-obesity effects in a high-fat diet-induced mouse model through the regulation of lipid metabolism (Ban et al., 2023). *Bifidobacterium lactis* suppresses adipocyte differentiation and lipid accumulation by downregulating the expression of lipogenic enzymes. Additionally, *Bifidobacterium lactis* administration reduces body weight and adipose tissue mass in mice, improves serum lipid profiles (Ban et al., 2023).

Some findings suggest that probiotics may improve obesity, hyperlipidemia, low-grade chronic inflammation, and obesity-associated liver damage, potentially through the modulation of antioxidant capacity and lipid metabolism. *Lactobacillus fermentum* reduces obesity, inflammation, and dyslipidemia in high-fat diet-induced obese mice through the regulation of antioxidant activity and lipid metabolism. *Lactobacillus fermentum* effectively mitigates high-fat diet-induced weight gain in mice, reduces adipocyte fat accumulation, and alleviates hepatocyte damage. It decreases levels of total cholesterol, LDL-C, and triglycerides while increasing HDL-C. Additionally, it increases hepatic mRNA expression of peroxisome proliferator-activated receptor (PPAR)-α and lipoprotein lipase, while downregulating PPAR-γ expression (Wu et al., 2021). In a high-fat diet-induced obese mouse model, treatment with *Lactococcus lactis* significantly reduced body weight and fat mass while markedly lowering serum levels of total cholesterol, triglycerides, and LDL-C. Notably, the expression levels of fatty acid synthase and PPAR-γ were significantly decreased in adipose tissue, indicating a potential molecular mechanism for the anti-obesity effects of probiotics. Additionally, histological examination showed a significant reduction in adipocyte size in the probiotics treatment group, indicating effective adipose tissue remodeling (Jeong et al., 2023). The results indicate that probiotics mitigate obesity by influencing essential molecular markers and altering lipid profiles.

*Lactobacillus plantarum* modulates lipid metabolism in C57BL/6 mice induced by a high-fat diet. *Lactobacillus plantarum* mitigates weight gain in obese mice, reduces hepatic lipid accumulation, and inhibits adipocyte hypertrophy. Additionally, it also lowers lipid levels in both serum and liver (Gan et al., 2020). *Lactobacillus plantarum* may mitigate lipid disorders related to excessive energy intake by reducing fat accumulation. This effect is mediated through the enhancement of cholesterol metabolism and fat clearance, thus aiding in lipid regulation in obese mice. The combination of probiotics *Lactobacillus plantarum* and *Lactobacillus curvatus* modulates hepatic lipid metabolism and mitigates diet-induced obesity. Both single-strain and multi-strain probiotics significantly reduce fat accumulation in adipose tissue and the liver. Single-probiotic strains reduce plasma and liver cholesterol levels, while combinations of multiple strains demonstrate enhanced effects on liver triglycerides. The experimental results also demonstrate that the probiotic combination more effectively suppresses the expression of genes involved in hepatic fatty acid synthesis, and simultaneously reduces the activity and expression of enzymes related to fatty acid oxidation (Yoo et al., 2013). Therefore, multi-strain probiotics may offer greater benefits than single-strain probiotics in mitigating fat accumulation and metabolic alterations associated with diet-induced obesity.

*Lactobacillus rhamnosus*, *Lactobacillus plantarum*, and *Lactobacillus brevis* can modulate lipid metabolism through the gut microbiota, contributing to weight loss. The results demonstrated that probiotics reduced body weight and blood glucose levels in obese mice and improved their lipid profiles. Furthermore, probiotics intervention led to significant enrichment in steroid hormone. *Lactobacillus rhamnosus* significantly enhanced the expression of genes related to arachidonic acid metabolism, bile secretion, and linoleic acid metabolism. *Lactobacillus brevis* repressed genes within the PPAR signaling pathway, whereas *Lactobacillus plantarum* activated genes involved in linoleic acid metabolism. Additionally, probiotics lowered the *Firmicutes*/*Bacteroidetes* ratio, a gut microbial composition associated with obesity. Probiotics significantly enhanced lipid-related pathways, promoting the biosynthesis of xanthophylls, prostaglandins, and fatty acid amides. Among these, *Lactobacillus rhamnosus* showed the greatest effect in alleviating obesity syndrome (Wu et al., 2023b). These findings suggest distinct regulatory patterns among different probiotic strains. However, the study only validated the effects of individual strains, leaving the potential impact of mixed strains on lipid metabolism and obesity reduction to be explored in future research.

### 2.3 Anti-inflammatory effect

Obesity is linked to chronic low-grade inflammation, which in turn elevates the risk of metabolic syndrome over time (Vitseva et al., 2008). Inflammatory factors within adipose tissue are linked to the mechanisms of obesity-related metabolic dysfunction, with elevated levels of C-reactive protein, TNF-α, and interleukin (IL)-6 accelerating the progression of chronic metabolic diseases in obesity (Vitseva et al., 2008; Zhao, 2013). Recent reports suggest that probiotics may mitigate obesity by modulating inflammatory factors. *Lactobacillus paracasei* is effective in alleviating obesity by modulating inflammatory factors and improving metabolic health. In obese mice, it reduces weight gain, liver fat accumulation, and levels of triglycerides, total cholesterol, and LDL cholesterol (Sun et al., 2023). Furthermore, this probiotic treatment reduces the expression of fatty acid synthase and inflammatory mediator IL-1β (Sun et al., 2023). These findings suggest that probiotics alleviate obesity by modulating gut microbiota and regulating the expression of genes involved in lipid synthesis and pro-inflammatory cytokines. *Lacticaseibacillus paracasei* exerts anti-obesity effects in rats by mitigating inflammation and insulin resistance. The administration of *Lacticaseibacillus paracasei* significantly reduced weight gain, liver weight, adipose tissue mass, and the enlargement of white adipocytes in obese rats. The abnormal lipid profiles in obese rats were normalized following *Lacticaseibacillus paracasei* treatment. Supplementation with *Lacticaseibacillus paracasei* attenuated chronic low-grade inflammation, evidenced by reduced serum levels of lipopolysaccharides and monocyte chemoattractant protein-1 (MCP-1), decreased macrophage infiltration in adipose tissue, and elevated serum adiponectin levels. Additionally, *Lacticaseibacillus paracasei* significantly reversed the upregulation of pro-inflammatory cytokine genes and the suppression of PPAR-γ mRNA in adipose tissue. *Lacticaseibacillus paracasei* intake led to a marked improvement in insulin resistance, as indicated by downregulated serum leptin levels. Consumption of *Lacticaseibacillus paracasei* also mitigated hepatic steatosis and significantly reduced the expression of hepatic lipogenic genes (Wu et al., 2023a).

*Lactobacillus fermentum* is particularly effective in reducing pro-inflammatory cytokines such as IL-1β, TNF-α, IL-6, and interferon-γ while increasing anti-inflammatory cytokines IL-10 and IL-4. It also lowers the activities of alanine aminotransferase and alkaline phosphatase, and enhances the mRNA expression of catalase and glutathione peroxidase (Wu et al., 2021). *Lactobacillus reuteri* attenuates weight gain, decreases food consumption, and limits fat accumulation in mice. It also modulates the gut microbiota, elevates IL-10, decreases IL-6 and TNF-α, and reduces liver injury, further improving insulin sensitivity and regulating lipid metabolism abnormalities (Yang et al., 2021). These findings suggest that *Lactobacillus reuteri* significantly ameliorates obesity by alleviating inflammation. *Bifidobacteria* can effectively mitigate obesity-associated inflammation and insulin resistance by modulating gut flora, and *Bifidobacterium pseudostreptococcus*, in particular, has shown promise in improving body mass index and reducing inflammation in children. Markers associated with inflammation, including circulating high-sensitivity C-reactive protein and MCP-1, were significantly reduced, while levels of HDL-C and omentin-1 increased (Sanchis-Chordà et al., 2019). *Bifidobacterium* supplementation ameliorates hepatic lipid accumulation, reduces inflammation in liver and adipose tissues, and enhances intestinal barrier function. These effects are associated with improvements in insulin sensitivity and metabolic endotoxemia (Ma et al., 2022).

## 3 The probiotics in adolescent obesity

**Figure 2:**
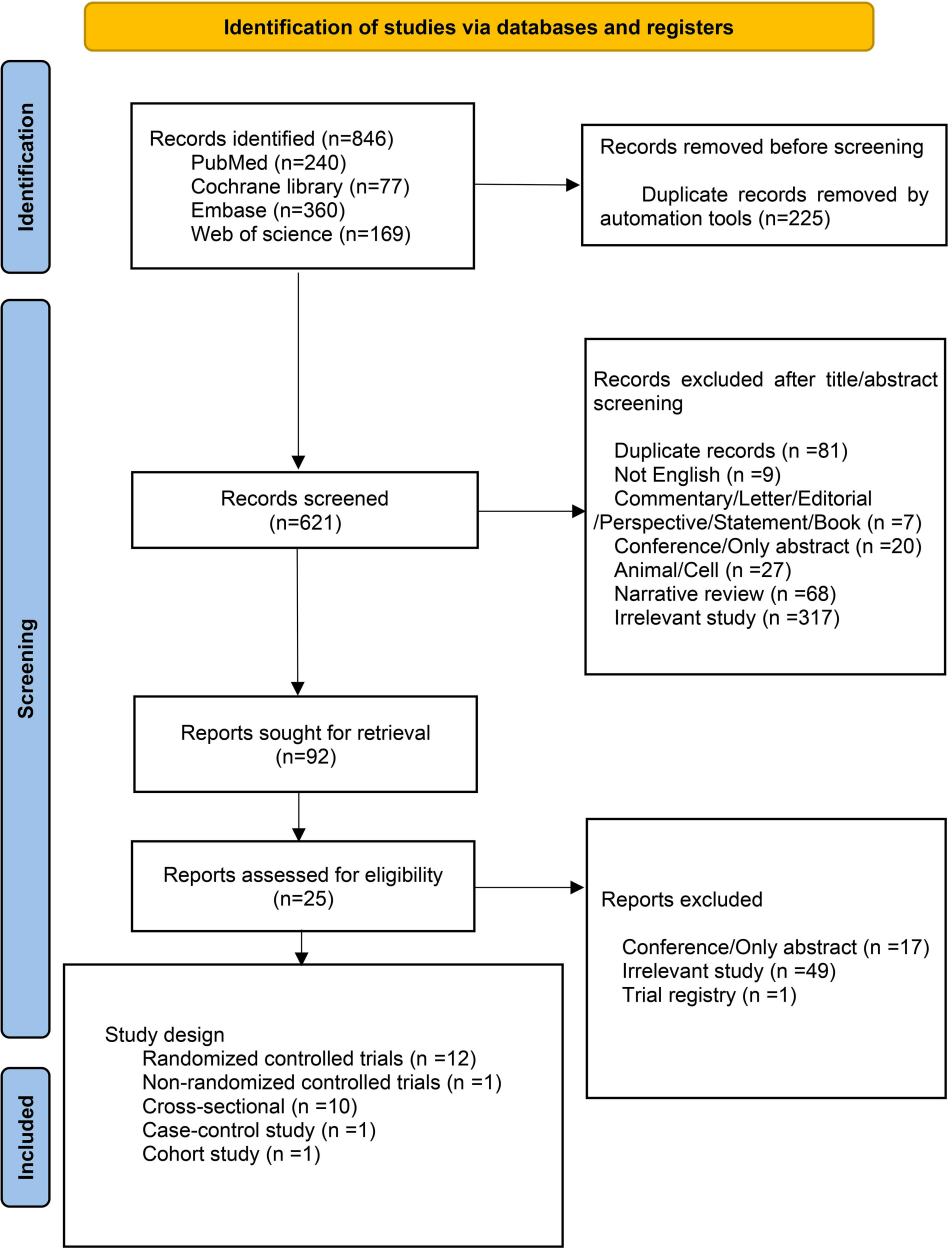
Flow diagram of study identification, screening, and inclusion in the systematic review.

In rodent models, the time required for the development of obesity usually exceeds the prepubertal period, which complicates the applicability and interpretation of findings involving adolescent animal models. We synthesized the clinical research findings on the effects of probiotics on weight control in adolescents. We conducted a literature search using PubMed, Cochrane, Embase, and Web of Science for articles published from inception to April 2024. The keywords and MeSH terms used in the search strategy included adolescent, overweight, obese, obesity, and probiotics. The full search strategies used in **Supplementary Table S1**. The study included obese or overweight adolescents undergoing probiotic treatment. Exclusion criteria were animal or *in vitro* studies, articles not written in English, and conference papers, abstracts, or reviews. **Figure 2** outlines the study selection process. There were 846 studies relevant to the search strategy, of which 225 were duplicates. Initial screening of titles and abstracts excluded 621 irrelevant documents. A detailed review of the remaining 92 documents further excluded 67 that did not meet the inclusion criteria, resulting in the final inclusion of 25 documents, 12 of which were RCTs.

Multiple clinical studies suggest that probiotics may contribute to alleviating obesity in adolescents. A prospective analysis revealed that probiotic treatment reduced MCP-1 levels and increased HDL-C and omentin-1 levels in obese Spanish adolescents. Single-strain probiotics intake may ameliorate the inflammatory state and reduce atherosclerotic markers in adolescents with obesity and insulin resistance (Sanchis-Chordà et al., 2019). A cross-over, double-blind, placebo-controlled clinical trial conducted in Finland found that short-term probiotic intervention has a beneficial effect on insulin metabolism in obese adolescents undergoing dietary training. All participants exhibited improved metabolic parameters, with reductions observed in both body weight and *Escherichia coli* levels (Solito et al., 2021). In addition, mixed probiotics had a significant effect on liver enzyme levels and ultrasound detection of fatty liver in adolescents. A randomized triple-blind placebo-controlled trial in Iran showed that a notable decrease in the mean levels of liver enzymes, specifically alanine aminotransferase (ALT) and aspartate aminotransferase (AST), in the group receiving probiotic treatment compared to the placebo group (Famouri et al., 2017). Additionally, probiotic treatment significantly improved non-alcoholic fatty liver disease and lipid profiles in obese adolescents. In comparison to the placebo group, adolescents in the probiotic group exhibited reductions in average cholesterol, LDL-C, triglycerides, and waist circumference (Famouri et al., 2017). In this study, the probiotic compound significantly affected waist circumference but had no impact on body weight or BMI. This may be due to the lack of strict enforcement of exercise and dietary control among adolescents by the researchers. Additionally, the three-month intervention may only have influenced serum markers, while longer-term intervention might be required to effectively impact body weight. A randomized, double-blind, placebo-controlled pilot clinical trial in obese adolescents in the United States revealed that probiotic therapy improves fasting blood glucose levels and reduces the *Firmicutes*-to-*Bacteroidetes* ratio in the gut microbiota (Verma et al., 2021). In a pilot randomized, controlled, double-arm trial conducted in Greece, the administration of a multi-strain probiotic intervention in overweight/obese adolescents with prediabetes showed subtle differences in glycemic control and gut homeostasis. Notably, after one month of probiotic supplementation, fasting glucose levels in the intervention group were significantly lower than those in the control group (Stefanaki et al., 2019). These findings suggest that a multi-strain probiotic intervention may modestly improve glycemic control and gut health in obese adolescents.

Moreover, researchers have identified that synbiotic treatment may also be beneficial in addressing obesity in adolescents. A randomized triple-blind controlled trial conducted in Iran revealed a significant reduction in BMI and waist circumference among obese adolescents treated with synbiotic compared to the control group (Kelishadi et al., 2014). Simultaneously, levels of inflammation-related markers TNF-α and IL-6 showed a significant reduction. These findings suggest that probiotics may play a beneficial role in managing inflammation, potentially contributing to obesity prevention and control. A randomized double-blind controlled trial in Turkey examined the impact of synbiotic on gut microbiota composition in obese adolescents. After 12 weeks of synbiotic intervention, the *Firmicutes*/*Bacteroidetes* ratio in the gut microbiota of obese adolescents decreased, accompanied by a reduction in BMI (Kilic Yildirim et al., 2023).

A Study from Ruijin Hospital affiliated with Shanghai Jiao Tong University School of Medicine have revealed for the first time that intestinal flora plays an important role in regulating host health and energy metabolism, and intestinal flora imbalance is closely related to obesity and diabetes. AKK bacteria are symbiotic bacteria that colonize the intestinal mucus layer, accounting for 1% to 5% of the intestinal microbiota. The research team isolated a new strain of AKK bacteria (AKK-WST01) with intellectual property rights from the feces of healthy Chinese people. The research results showed that the metabolic benefits of AKK-WST01 supplementation were closely related to the AKK bacteria abundance in the subjects’ baseline intestines, showing a high colonization rate in patients with low baseline AKK bacteria levels in the intestines, and significantly improved the patients’ weight, visceral fat, glucose and lipid metabolism and other indicators. In addition, fat oxidation was also significantly increased. Based on this, the research team proposed a new concept of personalized and precise supplementation of probiotics guided by the basic level of gut microbiota (Jiang, 2025).

Another study found that if a dominant pathogenic bacterium, Enterobacter cloacae B29, was isolated from the intestine of a severely obese patient weighing up to 175 kg and inoculated separately into sterile mice, it could cause severe obesity phenotype, and by taking probiotics to regulate the intestinal microbiota, their weight decreased by 51.4 kg (Zhao, 2015). Liu Xincai used molecular biology techniques such as PCR-DGGE and advantageous band cloning sequencing to establish an HFA animal model in obese Kazakh children using sterile C57BL/6J mice. The study showed that the gut microbiota is directly related to the occurrence of obesity (Liu et al., 2016).

After administering symbiotic bacteria (Lactobacillus acidophilus, Lactobacillus rhamnosus, Bifidobacterium bifidum, Bacillus subtilis, etc.), fructooligosaccharides (FOS), lactulose, and vitamins (A, B1, B2, B6, E, C) significantly reduced the body mass and BMI of patients (Ipar N et al., 2015). Research has found that compared to the control group, obese children and adolescents who were supplemented with probiotic mixtures (Lactobacillus casei, xylose, Lactobacillus thermophilus, Lactobacillus brevis, Lactobacillus acidophilus, Lactobacillus longum, and Lactobacillus bulgaricus, etc.) and prebiotics (vitamins A, C, and E) had significantly reduced body mass, BMI, and waist circumference (Kelishadi et al., 2014).

A single-center, prospective, randomized, double-blind, placebo-controlled clinical study on obese adolescents in Turkey found that daily intake of synbiotic, combined with diet and exercise, led to more significant improvements in anthropometric parameters (weight, BMI, waist circumference, and waist-to-height ratio) compared to the placebo (Yildirim et al., 2022).

However, some evidence suggests that probiotics may not effectively address obesity in adolescents. A double-blind randomized trial in Denmark found that a single probiotic intervention had no effect on metabolic syndrome markers related to obesity in adolescents. The study by Gobel et al. suggests that probiotics have no effect on obese adolescents (Gobel et al., 2012). In a double-blind, RCTs among obese Hispanic adolescents, probiotic supplementation led to weight gain without significant effects on liver fat or gut microbiota. The probiotic compound administration in obese adolescents significantly increased total and trunk fat content, without affecting liver fat/fibrosis, insulin/glucose levels, gut microbiota abundance, or gut hormones (Jones et al., 2018). This study suggests that probiotic treatment may contribute to adolescent obesity; however, the small sample size of 19 participants may introduce potential bias. Additionally, probiotic supplementation responses may vary among adolescents with differing obesity statuses and/or racial backgrounds. A randomized double-blind controlled trial from Denmark found an increase in the Bacteroides to *Firmicutes* ratio in the gut microbiota of obese adolescents following probiotic intake (Larsen et al., 2013). A non-randomized double-blind trial found that probiotic supplementation did not effectively promote weight loss or improve body composition in Brazilian adolescents. However, an exception was noted in adolescent males in the probiotic group, who exhibited a greater reduction in BMI (Marcelo et al., 2022). It is important to consider that the trial also included nutritional education and exercise interventions, making it difficult to exclude the influence of diet and physical activity. Additionally, as adolescent males are in a phase of muscle growth, this factor could also contribute to the observed decrease in BMI.

Based on the available clinical evidence, the role of probiotics in obese adolescents remains inconclusive. The evidence indicates that there are limited clinical trials involving probiotics in this population, with short intervention durations, typically around three months, and insufficient sample sizes (**Table 1**). However, based on the limited evidence available, it can be inferred that probiotics may have a positive impact on improving obesity in adolescents. The effect of combined probiotics might be more effective than that of a single strain. Additionally, this impact could be enhanced when combined with dietary and exercise interventions.

**Table 1:** Characteristics of included articles examining the effects of probiotic and symbiotic interventions on obesity-related parameters in adolescents.

Study (year)	Country	Design	Duration	N	Age	Intervention	Strain type and dosage	Conclusion	Reference
Famouri (2017)	Iran	RCT	12 weeks	64 (female/male: 32/32)	12.7 ± 1.95	multi-strain probiotics	*Lactobacillus acidophilus*, 3×10^9^ CFU; *Bifidobacterium lactis*, 6×10^9^ CFU; *Bifidobacterium bifidum*, 2×10^9^ CFU; *Lactobacillus rhamnosus*, 2×10^9^ CFU	Probiotics contribute to the improvement of liver enzyme levels in obese adolescents and are beneficial for alleviating fatty liver and lipid profiles.	(Famouri et al., 2017)
Gobel (2012)	Denmark	RCT	12 weeks	50 (female/male: 28/22)	13.1 ± 1.07	single-strain probiotics	*L salivarius* Ls-33	The probiotic intervention showed no detectable impact on inflammatory markers or metabolic syndrome parameters in obese adolescents with low-grade systemic inflammation.	(Gobel et al., 2012)
Jones (2018)	Spain	RCT	16 weeks	20 (female/male: 8/12)	14.7 ± 1.98	multi-strain probiotics	VSL#3^®^	Probiotics supplementation may increase adiposity in obese Latino adolescents without significantly affecting gut microbiota, appetite-regulating hormones, liver fat, fibrosis, or dietary intake.	(Jones et al., 2018)
Kelishadi (2014)	Iran	RCT	8 weeks	56	10.4 ± 2.24	synbiotics	*Lactobacillus casei*, *Lactobacillus rhamnosus*, *Streptococcus thermophilus*, *Bifidobacterium breve*, *Lactobacillus acidophilus*, *Bifidobacterium longum*, *Lactobacillus bulgaricus*, prebiotics	The supplementation with synbiotics exhibits a beneficial effect on inflammatory markers in obese adolescents.	(Kelishadi et al., 2014)
Kilic Yildirim (2023)	Turkey	RCT	12 weeks	54	8-17	synbiotics	*Lactobacillus acidophilus*, *Lacticaseibacillus. rhamnosus*, *Bifidobacterium bifidum*, *Bifidobacterium longum*, *Enterococcus faecium*, fructo-oligosaccharides	Synbiotic treatment is linked to alterations in gut microbiota composition and BMI reductions.	(Kilic Yildirim et al., 2023)
Larsen (2013)	Denmark	RCT	12 weeks	50	12-15	single-strain probiotics	*L. salivarius* Ls-33, 10^10^ CFU	Probiotics may alter the fecal microbiota of obese adolescents independently of metabolic syndrome.	(Larsen et al., 2013)
Marcelo (2022)	Brazil	non-RCT	6 months	44 (female/male: 19/25)	11.0 ± 2.27	single-strain probiotics	*Lactobacillus rhamnosus*, 1.2 ×10^8^ CFU	Supplementation with probiotics was ineffective in promoting weight loss or improving body composition in obese adolescents.	(Marcelo et al., 2022)
Sanchis-Chordà (2019)	Spain	RCT	13 weeks	48 (female/male: 24/24)	12.5 ± 2.62	single-strain probiotics	*Bifidobacterium. pseudocatenulatum*, between 1×10^9^ and 1×10^10^ CFU	Probiotic supplementation can improve the inflammatory status in obese adolescents with insulin resistance.	(Sanchis-Chordà et al., 2019)
Solito (2021)	Finland	RCT	8 weeks	101 (female/male: 54/47)	11.9 ± 2.71	single-strain probiotics	*Bifidobacterium breve*	Probiotic interventions effectively enhance insulin sensitivity and support weight loss in obese adolescents.	(Solito et al., 2021)
Stefanaki (2019)	Greece	RCT	4 months	17	13.5-15	multi-strain probiotics	*Streptococcus thermophilus*, *Bifidobacteria breve*, *Bifidobacteria longum*, *Bifidobacteria infantis*, *Lactobacillus acidophilus*, *Lactobacillus plantarum*, *Lactobacillus paracasei*, *Lactobacillus delbreuckii subspecies bulgaricus*	Probiotic intervention can moderately improve glycemic control and gut health in obese adolescents.	(Stefanaki et al., 2019)
Verma (2021)	USA	RCT	12 weeks	15 (female/male: 7/8)	15.9 ± 1.7	multi-strain probiotics	Visbiome^®^	Probiotics influence gut microbiota composition and may improve fasting glucose levels in obese adolescents compared to placebo.	(Verma et al., 2021)
Yildirim (2022)	Turkey	RCT	12 weeks	61 (female/male: 28/33)	8-17	synbiotics	*Lactobacillus acidophilus*, *Lacticaseibacillus rhamnosus*, *Bifidobacterium bifidum*, *Bifidobacterium longum*, *Enterococcus faecium*, and fructo-oligosaccharides	While adhering to dietary and exercise recommendations, the intake of probiotics can reduce weight, BMI, waist circumference, and hip circumference in obese adolescents.	(Yildirim et al., 2022)

## 4 Conclusion

The incidence of adolescent obesity is steadily increasing worldwide, emerging as a significant public health concern. Obesity is not merely an issue of excess body weight; it is closely associated with the development of various metabolic disorders, such as type 2 diabetes, hypertension, and cardiovascular diseases. Therefore, identifying effective interventions to slow or reverse the trend of obesity in adolescents is of critical importance. In this context, probiotics have gained substantial attention in academic circles as a potential intervention due to their ability to modulate the gut microbiota.

Current research indicates that the gut microbiota plays a crucial role in the onset and progression of obesity. Dysbiosis of the gut microbiota is strongly linked to metabolic abnormalities in the host, including imbalances in energy intake and expenditure and increased fat storage. Probiotics may improve the host metabolic status and reduce fat accumulation by regulating gut microbiota, promoting the growth of beneficial bacteria, and inhibiting the proliferation of harmful bacteria. Preliminary evidence from RCTs suggests that certain probiotic strains, such as *Bifidobacterium* and *Lactobacillus*, show promise in improving weight management and metabolic health in adolescents.

However, despite the scientific basis provided by existing studies for probiotic interventions in adolescent obesity, several limitations persist. For instance, many RCTs have small sample sizes, short intervention durations, and are often single-center studies, which raises concerns about the generalizability and applicability of the results. Additionally, the effects of different probiotic strains vary significantly, and questions regarding the optimal strain selection, dosage, and treatment duration remain unresolved. Thus, further large-scale, multicenter, and long-term follow-up studies are needed to confirm the efficacy and safety of probiotics in adolescent obesity interventions.

Therefore, probiotics may become a component of comprehensive obesity treatment strategies, particularly when combined with traditional methods such as dietary adjustments and exercise interventions. As research progresses, there is reason to believe that probiotics could provide a safe, effective, and well-tolerated adjunctive treatment for adolescent obesity. However, more scientific evidence is needed to determine their applicability across diverse populations and their long-term effects, thereby providing a stronger foundation for clinical practice.
