# Supplementary material for: The role of probiotics in adolescents’ obesity
Source: Front Cell Infect Microbiol. 2025 Jul 2;15:1546627. doi: 10.3389/fcimb.2025.1546627 (PMC12263623; doi:10.3389/fcimb.2025.1546627)
Supplement: Supplementary file 1 [file Table1.docx]

**The role of probiotics in adolescents’ obesity**

Xiao-ping Chen ^a^, Li You ^a^, Yong Jia ^a*^.

^a^ College of Physical Education and Health, Chongqing College of International Business and Economics, Chongqing 401520, China.

[Table S1 Search strategy (updated on April 17, 2024). 1](#_Toc185252262)

[Table S1-1. Search strategy in PubMed. 1](#_Toc185252263)

[Table S1-2. Search strategy in Cochrane library. 3](#_Toc185252264)

[Table S1-3. Search strategy in Embase. 4](#_Toc185252265)

**Table S1 Search strategy (updated on April 17, 2024).**

**Table S1-1. Search strategy in PubMed.**

| Process | Search terms | Notes | No. of results |
| --- | --- | --- | --- |
| #1 | "Adolescent"[Mesh Terms] OR "adolescent*" [tiab] OR "teen*" [tiab] | adolescent | 2,329,722 |
| #2 | "Overweight"[MeSH Terms] OR "Overweight" [tiab] OR "obese" [tiab] OR "obesity" [tiab] | obesity | 475,530 |
| #3 | #1 AND #2 |  | 70,086 |
| #4 | (("Probiotics" [Mesh] OR "Lactobacillus" [Mesh] OR "Lactococcus" [Mesh] OR "Bifidobacterium" [Mesh] OR "Propionibacterium" [Mesh] OR "Streptococcus" [Mesh] OR "Bacteroides" [Mesh]) OR "Probiotic*" OR "VSL 3" [tiab] OR "lactobacillus" [tiab] OR "lactococcus" [tiab] OR "bifidobacterium" [tiab] OR "propionibacterium" [tiab] OR "streptococcus" [tiab] OR "Bacteroides" [tiab]) | probiotics | 233,518 |
| #5 | #3 AND #4 |  | 240 |

**Table S1-2. Search strategy in Cochrane library.**

| Process | Search terms | Notes | No. of results |
| --- | --- | --- | --- |
| #1 | MeSH descriptor: [Adolescent] explode all trees OR (adolescent):ti,ab,kw OR (teen*):ti,ab,kw 3589 | adolescent | 162774 |
| #2 | MeSH descriptor: [Overweight] explode all trees OR (Overweight):ti,ab,kw OR (obese):ti,ab,kw OR (obesity):ti,ab,kw | obesity | 60381 |
| #3 | #1 AND #2 |  | 6310 |
| #4 | ((MeSH descriptor: [Probiotics] explode all trees OR MeSH descriptor: [Lactobacillus] explode all trees OR MeSH descriptor: [Lactococcus] explode all trees OR MeSH descriptor: [Bifidobacterium] explode all trees OR MeSH descriptor: [Propionibacterium] explode all trees OR MeSH descriptor: [Streptococcus] explode all trees OR MeSH descriptor: [Bacteroides] explode all trees) OR (Probiotic*):ti,ab,kw OR (VSL 3):ti,ab,kw OR (lactobacillus):ti,ab,kw OR (lactococcus):ti,ab,kw OR (bifidobacterium):ti,ab,kw OR (propionibacterium):ti,ab,kw OR (streptococcus):ti,ab,kw OR (Bacteroides):ti,ab,kw) | probiotics | 19057 |
| #5 | #3 AND #4 |  | 77 |

**Table S1-3. Search strategy in Embase.**

| Process | Search terms | Notes | No. of results |
| --- | --- | --- | --- |
| #1 | 'adolescent'/exp OR 'adolescent*':ab,ti OR 'teen*':ab,ti | adolescent | 2,077,098 |
| #2 | 'obesity'/exp OR 'overweight':ab,ti OR 'obese':ab,ti OR 'obesity':ab,ti | obesity | 827,624 |
| #3 | #1 AND #2 |  | 78,629 |
| #4 | (('probiotic agent'/exp OR 'Lactobacillus'/exp OR 'Lactococcus'/exp OR 'Bifidobacterium'/exp OR 'Propionibacterium'/exp OR 'Streptococcus'/exp OR 'Bacteroides'/exp) OR 'Probiotic*':ab,ti OR 'VSL 3':ab,ti OR 'lactobacillus':ab,ti OR 'lactococcus':ab,ti OR 'bifidobacterium':ab,ti OR 'propionibacterium':ab,ti OR 'streptococcus':ab,ti OR 'Bacteroides':ab,ti) | probiotics | 337,594 |
| #5 | #3 AND #4 |  | 360 |

**Table S1-4. Search strategy in Web of science.**

| Process | Search terms | Notes | No. of results |
| --- | --- | --- | --- |
| #1 | ((TS=(Adolescent)) OR TS=(adolescent*)) OR TS=(teen*) | adolescent | 555,019 |
| #2 | (((TS=(Overweight)) OR TS=(Overweight)) OR TS=(obese)) OR TS=(obesity) | obesity | 547,675 |
| #3 | #1 AND #2 |  | 46,855 |
| #4 | ((((((((((((((TS=(Probiotics)) OR TS=(Lactobacillus)) OR TS=(Lactococcus)) OR TS=(Bifidobacterium)) OR TS=(Propionibacterium)) OR TS=(Streptococcus)) OR TS=(Bacteroides)) OR TS=(Probiotic*)) OR TS=(VSL 3)) OR TS=(lactobacillus)) OR TS=(lactococcus)) OR TS=(bifidobacterium)) OR TS=(propionibacterium)) OR TS=(streptococcus)) OR TS=(Bacteroides) | probiotics | 258,052 |
| #5 | #3 AND #4 |  | 169 |
